# The IL-1*β* Receptor Antagonist SER140 Postpones the Onset of Diabetes in Female Nonobese Diabetic Mice

**DOI:** 10.1155/2016/7484601

**Published:** 2016-02-03

**Authors:** Helena Cucak, Gitte Hansen, Niels Vrang, Torben Skarsfeldt, Eva Steiness, Jacob Jelsing

**Affiliations:** ^1^Gubra ApS, Agern Alle 1, 2970 Hørsholm, Denmark; ^2^Serodus ASA, Gaustadalléen 21, 0349 Oslo, Norway

## Abstract

The cytokine interleukin-1*β* (IL-1*β*) is known to stimulate proinflammatory immune responses and impair *β*-cell function and viability, all critical events in the pathogenesis of type 1 diabetes (T1D). Here we evaluate the effect of SER140, a small peptide IL-1*β* receptor antagonist, on diabetes progression and cellular pancreatic changes in female nonobese diabetic (NOD) mice. Eight weeks of treatment with SER140 reduced the incidence of diabetes by more than 50% compared with vehicle, decreased blood glucose, and increased plasma insulin. Additionally, SER140 changed the endocrine and immune cells dynamics in the NOD mouse pancreas. Together, the data suggest that SER140 treatment postpones the onset of diabetes in female NOD mice by interfering with IL-1*β* activated pathways.

## 1. Introduction

T1D is characterized by progressive autoimmune/autoinflammatory destruction of pancreatic *β*-cells over a period of years, resulting in absolute insulin deficiency and the need for lifelong dependence on exogenous insulin administration. In addition, T1D increases the high risk of one or more acute and late disease-associated complications, for example, neuropathy, hypoglycemia, cardiovascular disease, and retinopathy [1, 2]. Initial diagnosis has been coupled to a substantial decrease (~90% loss) in *β*-cell mass [3], subsequently leading to a complete loss of insulin production. Hence, interventions that prevent or halt the predestined decline of *β*-cell function are needed.

Several clinical trials are aiming at immune intervention or modulation with the key goal to induce immune tolerance against *β*-cells and thereby prevent autoimmune destruction [4]. These trials have shown varied clinical efficacy but have to some extent provided insight into the role of the immune cell triggered *β*-cell death [5, 6]. Targeting the adaptive immune system to preserve *β*-cell function in new-onset T1D has shown temporary suppression of disease [7–9]. However, recently suppression of the innate arm of the immune system has been suggested to have even more beneficial effects [10]. In particular, much attention has focused on IL-1*β*, which is one of the primary innate proinflammatory cytokines shown to cause tissue damage and organ failure, hence being a key mediator in autoinflammatory conditions [11, 12]. IL-1*β* has been assigned a key role in T1D and has long been known to cause *β*-cell dysfunction and death [13]. IL-1*β* is produced and released by several cell types in response to tissue insult, or in the context of diabetes, by *β*-cells under hyperglycemic conditions [14]. Once present in the pancreatic microenvironment it can act locally to inhibit insulin synthesis and secretion and induce *β*-cell apoptosis through activation of proapoptotic JNK, MAPK, and NF*κ*B signaling pathways [13]. Additionally, IL-1*β* can drive T1D pathogenesis by enhancing the recruitment of immune cells and modify the adaptive immune response towards a more proinflammatory cell repertoire [12, 15]. Thus, there is a strong preclinical rationale for IL-1*β* antagonism to prevent or reverse T1D and T2D onset and IL-1*β* has become a promising target for therapeutic intervention [13, 16].

SER140 is a 10-amino-acid peptide IL-1*β* receptor antagonist that has previously been shown to inhibit interleukin-1*β*-induced NF*κ*B signaling and macrophage secretion of TNF-*α* and hence a potent inhibitor of inflammatory responses [17]. Further, SER140 exceeded the maximal effect of anakinra (a recombinant, nonglycosylated version of human IL-1R antagonist) in averting IL-1*β*-induced apoptosis in rat pancreatic islets [17] and is currently being evaluated for treatment of T2D. The many shared features between both major diabetes types justify similar efforts of interfering with IL-1*β* signaling in T1D. In this report, we took advantage of the NOD mouse model that spontaneously develops T1D [18]. The NOD mouse model seems to reflect several crucial aspects of the human disease including pancreatic inflammation [19]. Further, IL-1*β*R deficiency has been shown to reduce progression to diabetes in NOD mice [20] making this the ideal model to examine the potential beneficial effects of SER140 in T1D.

## 2. Materials and Methods

### 2.1. Animals

A total of 40 female NOD mice (8-9 weeks of age, Taconic (USA)) were transferred to the Gubra animal unit. The animals were group-housed (5 mice/cage) throughout the habituation and study period in a light-temperature- and humidity-controlled room with free access to food and water. All animal experiments were conducted in accordance with Gubra bioethical guidelines, which are fully compliant with internationally accepted principles for the care and use of laboratory animals. The described experiments are covered by personal licenses for Jacob Jelsing (2013-15-2934-00784) issued by the Danish Committee for Animal Research.

### 2.2. *In Vivo* Procedures

 Nonfasting blood glucose (BG) was monitored biweekly before the experiment start. On day 3, animals were randomized according to BG and then body weight into two groups: a vehicle group (QD) (*n* = 20) and a SER140 group, 10 mg/kg (QD) (*n* = 20). The compound was administered subcutaneously once daily. SER140 was provided by Phlogo ApS, Copenhagen, Denmark (5 mg/mL in water), and diluted in PBS at the required concentration for injection (10 mg/kg, S.C). Throughout the study, animals had ad libitum access to food and water. Body weight and food and water intake were recorded biweekly from arrival and throughout the study period. Samples for measuring nonfasting BG were collected biweekly from the tail vein. Animals were terminated on day 56 and BG was measured using a BIOSEN c-Line glucose meter (EKF Diagnostics, Germany), HbA1c using autoanalyzer Cobas C-111 with commercial kit (Roche Diagnostics, Germany), and insulin using ultrasensitive insulin ELISA (Mercodia, Sweden) according to the manufacturer's instructions.

### 2.3. Pancreas Preparation

**Figure 1:**
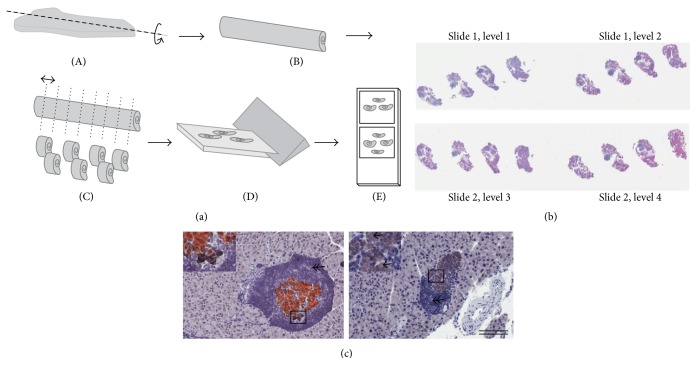
Schematic illustration of sampling principles for stereological assessment. (a) Formalin fixed pancreas samples were rolled tightly into strips of gauze, infiltrated in paraffin, and cut into 3-4 systematic uniform random tissue slabs with a razor blade fractionator and embedded in one paraffin block with the cut surface down. The blocks were trimmed and four sections for each animal were sampled 300 *μ*m apart and arranged on two glass slides (b), representing a systematic uniform random sample of the whole pancreas. (c) Representative images of pancreatic sections from untreated mice demonstrating *β*-cells (brown: insulin), non-*β*-cells (black: glucagon, somatostatin, and pancreatic polypeptide), unstained endocrine cells (arrow), and surrounding immune cells (double arrow: hematoxylin). Scale bars = 400 *μ*m.

The pancreas was removed, immersion-fixed in 4% formaldehyde at 4°C for 24 hrs, and processed as described previously [21]. Briefly, the pancreas was rolled into a cylinder, infiltrated with paraffin overnight using an automated Miles Scientific Tissue-TEK VIP Tissue Processor (Sakura), and cut into three to four systematic uniform random tissue slabs with a razor blade fractionator. The slabs were embedded on their cut surface in one paraffin block. The blocks were trimmed and three series of 4 *μ*m sections were sampled providing twelve to fifteen levels in total for quantitative analyses. One series of sections were subsequently subjected to standard hematoxylin staining for stereological assessment of immune cell infiltrates in combination with a double immunohistochemical staining procedure for stereological assessment of *β*- and non-*β*-cells (Figure 1). Other series were used for immunohistochemistry on specific immune cell populations and for expression of proinflammatory cytokines by* in situ* hybridization (ISH) (RNAscope, Advance Cell Diagnostics, China).

### 2.4. Immunohistochemistry

Antibodies and concentrations used are listed in Table 1. All stainings were performed on an Autostainer Link 48 (Dako) and finally digitized under a 20x objective in an Aperio ScanScope AT slide scanner for qualitative image analysis.

#### 2.4.1. *β*-/Non-*β*-Cells

After deparaffinization in a series of ethanol and xylene and antigen retrieval in citrate buffer (10 mM, pH 6) sections were quenched with 1% H_2_O_2_ in KPBS and blocked with SA-biotin kit (X0590, Dako) and 5% swine serum in TBS-T + 1% BSA, followed by incubation with the primary non-*β* antibody-cocktail. Sections were then incubated with the secondary biotinylated antibody (Fab2) fragment followed by SA-peroxidase (HRP) and visualized with diaminobenzidine and NiSO_4_. For *β*-cells, sections were blocked in 10% rabbit serum (X0902, Dako), stained with anti-insulin and HRP-secondary antibody. Finally, the sections were developed in NovaRed (SK4800, Vector Laboratories), stained in a Mayer solution, dehydrated, and mounted in Pertex. See Table 1.

#### 2.4.2. T-Cells/*β*-Cells and Macrophages

Deparaffinization was performed as previously followed by antigen retrieval in TRIS-EGTA buffer (pH 9) or by proteinase K treatment (F4/80). Endogenous peroxidase activity was quenched in 1% H_2_O_2_ and blocked 5% swine serum, 1% BSA, and 0.2% Tween 20. Sections were then incubated with primary antibodies (CD3, CD20, and F4/80) followed by corresponding secondary antibodies. Signal was amplified using Vectastain ABC amplification system (Vector Laboratories) (CD20) and Envision+ HRP-coupled polymer system (Dako) and visualized in a DAB solution. See Table 1.

### 2.5. Stereological Assessment of Cell Mass

The stereological estimation of cell mass was performed by an observer blinded to the experimental groups. The cell mass was estimated by point counting with all points hitting the structure of interest being counted. Sections were scanned in a random systematic way using the newCAST system (Visiopharm, Hørsholm, Denmark) to control the stage and collection of data. A single-point grid per frame was used to estimate pancreas mass and a denser grid was used to estimate *β*-cell/non-*β*-cell and immune cell mass. Similarly, the grid system was used to correct the presence of nonpancreatic elements in the dissected sample. In principle, the point grid is used to estimate the area fraction of counted cell types. The number of points hitting the structure of interest is then converted into mass by taking the grid ratio into consideration [22].

### 2.6. *In Situ* Hybridization

ISH was performed using the RNAscope 2.0 High Definition-RED Assay (Advanced Cell Diagnostics) with IL-6 (NM_031168.1), TNF-*α* (NM_013693), and IFN-*γ* (NM_008337.3) specific probes according to the manufacturer's instructions.

### 2.7. Statistical Analysis

Graphical presentations, calculations, and statistical analyses were carried out with GraphPad software. Statistical analyses were performed using a two-way ANOVA with repeated measures and Bonferroni post hoc analysis or unpaired Student's *t*-test. *p* < 0.05 was considered statistically significant.

## 3. Results

### 3.1. Postponed Diabetes Onset in SER140 Treated Mice

**Figure 2:**
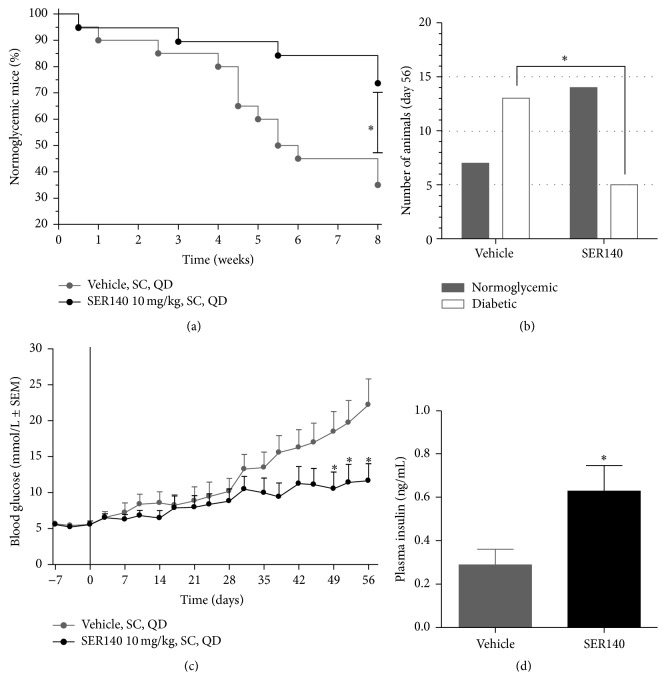
Development of diabetes in NOD mice. (a) Proportion of mice (percentile) having normal glucose levels defined as nonfasting BG below 10 mmol/L from day 0 to day 56. (b) Number of normoglycemic versus diabetic animals at day 56. Statistical significance was obtained between the variables (*p* = 0.025, Fisher's exact test) with a higher incidence of diabetes in the vehicle group ((a) and (b)). (c) Morning fed BG during a time period from day −7 to day 56. (d) Plasma insulin levels at day 56. Unpaired *t*-test ^*∗*^ *p* < 0.05. ((c) and (d)) Data are presented as mean ± SEM (*n* = 18–20/group). (c) Two-way ANOVA w/Bonferroni post hoc test. (d) Unpaired *t*-test. ((c) and (d)) ^*∗*^ *p* < 0.05 compared to vehicle.

SER140 was able to postpone the development of diabetes in NOD mice (Figure 2). The first incidence of diabetes (BG > 10 mmol/L) was observed in both treatment groups at experimental week one. However, in week two, a total of three incidences were observed in the vehicle group with no diabetic cases in the SER140 treated group (Figure 2(a)). Afterwards, the proportion of normoglycemic SER140 treated mice compared to vehicle became even more evident over time leading to a significantly lower proportion of diabetic mice in the SER140 group at termination (Figures 2(a) and 2(b)).

**Figure 3:**
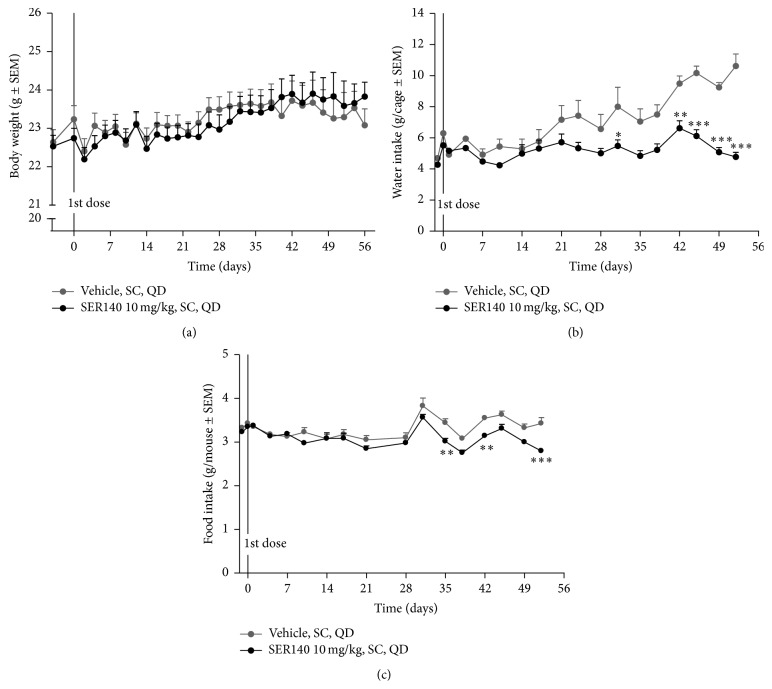
Daily body weight (a), water intake (b), and food intake (c) during the 56-day study period. All data are presented as mean ± SEM (*n* = 18–20/group). ((a)–(c)) ^*∗*^ *p* < 0.05, ^*∗∗*^ *p* < 0.01, and ^*∗∗∗*^ *p* < 0.001 compared to vehicle; two-way ANOVA w/Bonferroni post hoc test.

The SER140 group displayed significant reduction of mean BG as well as a significant increase in mean plasma insulin at the end of the study (Figures 2(c) and 2(d)) with a nonsignificant tendency towards decrease in HbA1c (6.05 ± 0.42 versus 5.09 ± 0.41, *p* = 0.11). No significant difference was observed in overall body weight (Figure 3(a)) whereas both food and water intake (measured as average per cage) decreased in the SER140 treated group (Figures 3(b) and 3(c)), most likely related to the increased number of diabetic animals in the untreated group.

One normoglycemic SER140 treated animal was excluded from the study at experimental day 28 due to general misbehavior unrelated to treatment and was excluded from all measurements. In addition, one vehicle mouse and one SER140 treated mouse died before study end, probably as a consequence of early onset of diabetes, and were not included in the histological analyses due to rapid tissue decay.

### 3.2. Preservative Role of SER140 in *β*-Cell Mass

**Figure 4:**
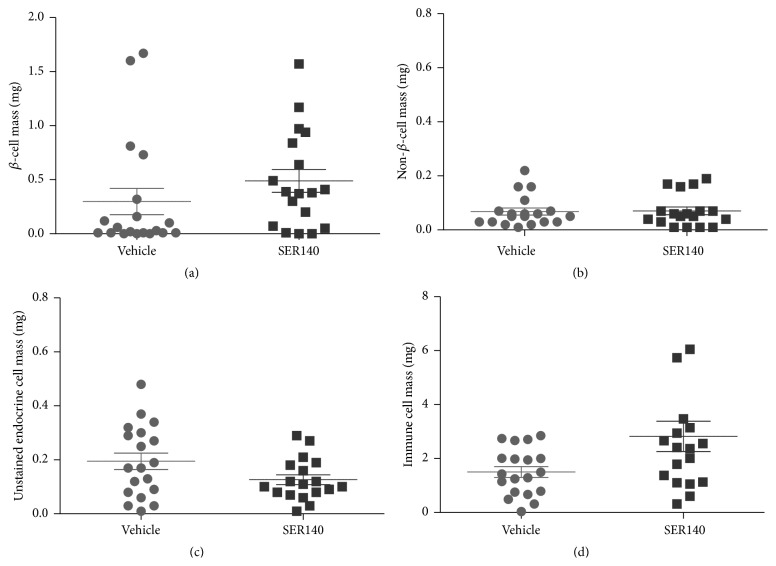
Stereological analyses of NOD mice pancreata. (a) *β*-cell mass, (b) non-*β*-cell mass, (c) unstained endocrine cell mass, and (d) immune cell mass in vehicle and SER140 treated NOD mice. The average for each group is marked by horizontal lines (*n* = 18-19/group).

**Figure 5:**
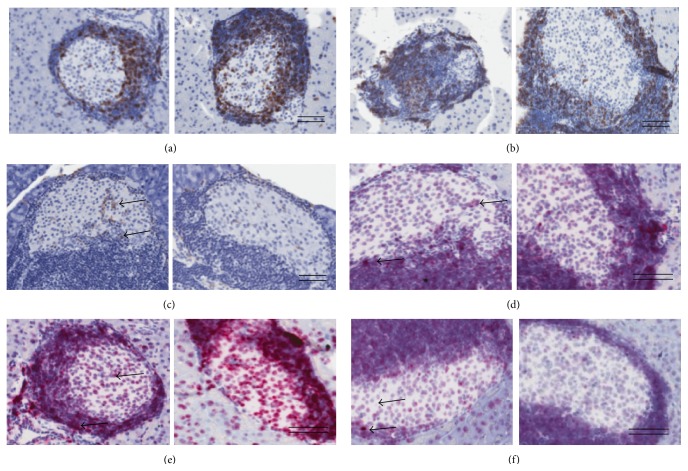
Immune cell distribution and expression of proinflammatory cytokines in NOD mice pancreata. Immunohistochemistry staining of the immune cells in pancreatic islets of vehicle (left) and SER140 treated (right) mice at day 56 of treatment, for (a) CD3, (b) CD20, and (c) F4/80. Arrows indicate F4/80^+^ cells.* In situ* hybridization analysis of the expression of genes encoding the proinflammatory cytokines IL-6 (d), TNF-*α* (e), and IFN-*γ* (f) in and around pancreatic islets (red dots, arrows) in vehicle versus SER140 treated mice at day 56 of treatment ((d)–(f)). ((a)–(f)) Left: vehicle, right: SER140 treated. Scale bars = 100 *μ*m.

The effect of SER140 on endocrine and immune cell mass was performed on systematic uniform random samples of the whole pancreas (Figures 1(a)–1(c)). Total pancreas mass (corrected for fat and lymphoid tissue) was slightly higher in SER140 treated mice compared to vehicle (272 ± 12.5 versus 313 ± 8.83, *p* = 0.012). The quantitative analyses of immunohistochemically stained islet cell types (Figure 1(c)) displayed a tendency towards increased *β*-cell mass with treatment although not significant (Figure 4(a)). No treatment related change was observed in non-*β*-cell mass (Figure 4(b)), whereas the mass of unstained endocrine cells (endocrine cells that have lost the expression of hormones) tended to be reduced following SER140 treatment (Figure 4(c), *p* = 0.065). Immune cell infiltrates, as identified by dense hematoxylin staining, were observed around islets in both groups (Figure 1(c)) but being significantly higher in SER140 treated mice (Figure 4(d)). The qualitative analysis of specific immune cell subsets revealed that immune cell infiltrates mainly consisted of *β*-cells (CD20^+^), T-cells (CD3^+^), and only few macrophages (F4/80^+^) but with no noticeable changes in immune cell subtypes with treatment (Figures 5(a)–5(c)). Moreover, consistent with insulitis, proinflammatory cytokines were observed around and within the islets (Figures 5(d)–5(f)) in both groups.

**Figure 6:**
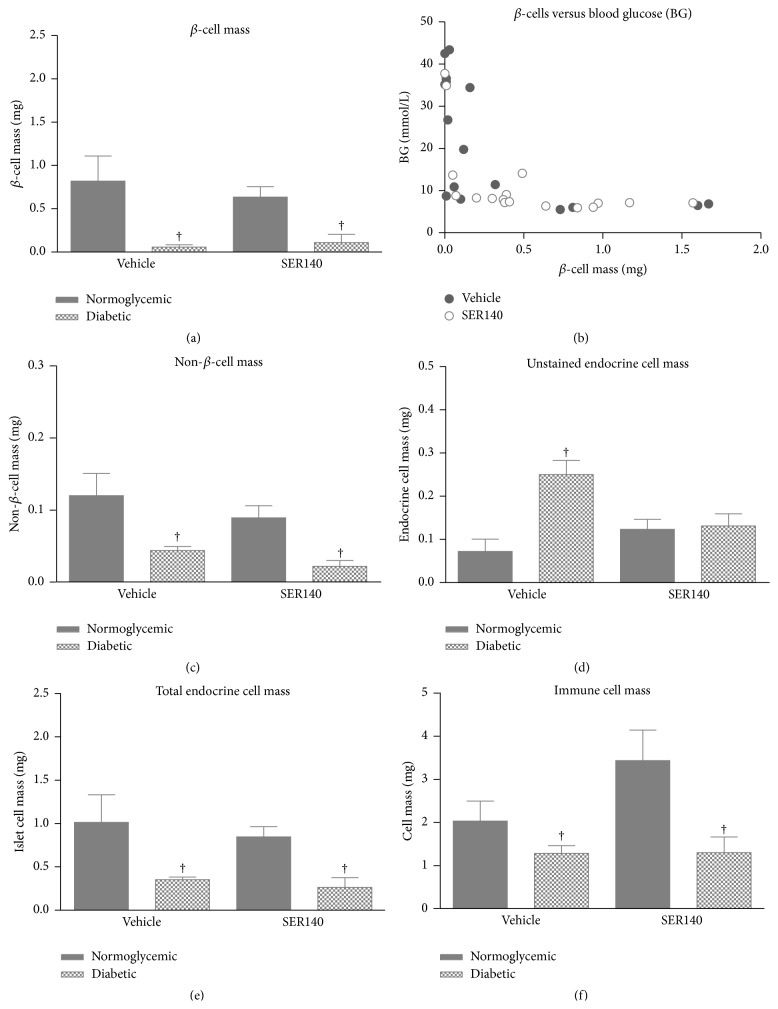
Stereological analyses of nondiabetic and diabetic NOD mice pancreata. (a) *β*-cell mass, (b) *β*-cell mass versus BG, (c) non-*β*-cell mass, (d) unstained endocrine cell mass, and (e) immune cell mass in diabetic and nondiabetic vehicle and SER140 treated NOD mice. (f) *β*-cell mass versus immune cell mass. ((a), (c), (d), and (e)) Data are presented as mean ± SEM (*n* = 18-19/group); one-way ANOVA with Turkey's multiple comparison test; ^†^ *p* < 0.05 versus vehicle normoglycemic mice.

A subgroup analysis of normoglycemic (BG < 10 mmol/L) and diabetic (BG > 10 mmol/L) animals revealed a higher *β*-cells mass in normoglycemic mice versus diabetic mice irrespectively of treatment (Figure 6(a), Table 2). However, further analyses of BG levels as a function of *β*-cells mass revealed that even a minute mass of enduring *β*-cells is able to compensate and maintain normal BG levels (Figure 6(b)). Non-*β*-cell mass was also significantly lower in the diabetic animals as compared to normoglycemic mice irrespectively of treatment demonstrating a progressive loss of non-*β*-cells in the NOD model with diabetes onset (Figure 6(c)). Subgroup analyses of the nonimmunoreactive endocrine cell masses revealed a significant effect of diabetes status and a significant treatment/diabetes interaction (Figure 6(d)). Collectively, the total endocrine cell pool was significantly reduced in diabetic mice (Figure 6(e)). Finally, immune cell mass was lower in diabetic mice compared to normoglycemic animals with no significant interaction or treatment effect (Figure 6(f)).

## 4. Discussion

The NOD mouse presents early signs of insulitis, spontaneously develops autoimmune diabetes, and is generally regarded as a suitable animal model of human T1D. Here we report that the novel IL-1*β*R antagonist SER140 is able to postpone the onset of diabetes in female NOD mice coupled to an overall decrease in BG levels and increased insulin levels upon treatment.

There is a robust justification for blocking IL-1*β* in diabetes. Recombinant forms of the naturally occurring IL-1*β* receptor antagonist have already proved to be efficacious in a broad spectrum of inflammatory diseases, including T2D [16]. However, two comprehensive clinical trials of IL-1*β* blockade in T1D have shown divergent results. Anakinra alone appeared to be ineffective in reversal of T1D in hyperglycemic NOD mice [23], whereas another study showed beneficial effects in a carefully selected group of long-standing T1D patients with a “type 2 phenotype,” suggesting that the compound is more effective in conditions of chronic hyperglycemia [24]. Further, preclinical studies targeting IL-1*β*R signaling in GK-rats [25], as well as in mice fed a high-fat/high-sucrose diet (HFD) [26, 27], resulted in significantly improved glucose control and *β*-cell function. However, despite an ample amount of preclinical and clinical studies a defined mechanism of action for IL-1*β* antagonism in diabetes is still lacking. IL-1*β* orchestrates several immunological and cellular pathways, hence proposing several mechanistic explanations for prodiabetic effect, due to either a modulatory role in the local immune system or a direct role in pancreatic *β*-cell function and/or survival [13]. In addition, questions remain regarding whether a beneficial effect of IL-1*β* antagonism on BG is a consequence of protection of *β*-cell mass* per se* or due to an overall improvement of glucose control (*β*-cell function and insulin sensitivity). Indeed, the potential of IL-1*β* antagonism to improve *β*-cell function has been reported in T2D patients [16], but this could also be explained by the T2D associated deficit in *β*-cell mass [28]. Cytotoxicity sufficient to induce *β*-cell apoptosis will presumably influence the function of surviving *β*-cells making a distinction of the relative contributions of these related processes nearly impossible.

In the present study, treatment related changes in serum insulin levels were partly reflected in the preserved *β*-cell mass* per se*. The assumption that SER140 treatment maintains functional *β*-cell mass is further supported by the decrease in nonimmunoreactive endocrine cell mass. The exact phenotype of the unstained endocrine cells is currently unknown, but it is speculated that they represent *β*-cells that have lost the capacity to produce insulin since an inverse correlation between *β*-cells and unstained endocrine cells was observed (data not shown).

Moreover, we found a proportion of normoglycemic mice having very low *β*-cell mass unrelated to treatment, indicating that SER140 does not evidently strengthen residual *β*-cell function. It appears that even small numbers of residual *β*-cells can compensate and maintain normal BG levels as supported by others demonstrating an effective insulin response despite massive loss of *β*-cells [29]. Hence, *β*-cell mass may be an inadequate determinant of overall *β*-cell function in this model. In addition, it is well known that some aging female NOD mice never progress to develop diabetes [30]. The compensatory capacity of a minor residual *β*-cell mass combined with the heterogeneity of disease penetrance in the NOD mice could be the explanation for the lack of statistical significance in the effect on *β*-cell mass upon SER140 treatment.

Our current results indicate that SER140 treatment did not reduce pancreatic immune cell mass. This finding partly contradicts an anti-inflammatory effect of SER140 in the pancreas but is in line with other studies showing that IL-1*β* antagonism does not alter the adaptive immune response [31]. Somewhat unexpected, the subgroup analyses of diabetic and nondiabetic mice demonstrated a significantly lower immune cell mass in diabetic animals and a conceivably higher immune cell mass in compound treated mice conflicting with an anti-inflammatory effect of SER140. However, if immune cell recruitment is increasing in the prediabetic stage with a subsequent decrease following diabetes onset (as indicated by the current data), the apparent higher immune cell mass in SER140 treated mice can simply be explained by a SER140 induced postponement of diabetes onset. More studies are, however, needed to accurately depict the endocrine and immune cell dynamics in the female NOD mouse model. Alternatively, the apparent higher immune cell mass could be explained by a reduced function of Treg cells within inflamed islets after disease onset in NOD mice [32] and that SER140 blockade of IL-1*β* signaling might have a preservative function on these cells [33, 34]. The delayed reduction of Treg cell function might also explain the significant increase in the plasma levels of the anti-inflammatory cytokine IL-10 that has been reported after SER140 treatment [17].

In accordance with previous studies [35, 36] we observed substantial immune cell infiltration into the pancreatic islets being mainly composed of lymphocytes as indicative of insulitis. Consistent with an inflamed pancreas, a substantial proinflammatory cytokine expression was observed around and within the islets. We did, however, not observe any apparent changes in the number of specific immune cell subsets or apparent difference in cytokine expression with treatment. It has previously been shown that even low concentrations of IL-1*β* can exert cytotoxic effect on pancreatic *β*-cells, partly due to their high density of IL-1*β* receptors compared to other cell types [13]. However, based on the data presented here it is not possible to differentiate between the effects of IL-1*β* blockade on the *β*-cells and on the immune system. Hence, based on these limitations we can only cautiously speculate that the *β*-cell sparing effect of SER140 is mediated through direct blocking of the proapoptotic action of IL-1*β* on *β*-cells.

## 5. Conclusion

We have shown that the IL-1*β*R antagonist SER140 at 10 mg/kg subcutaneously is able to prevent the destruction or damaging of the insulin producing *β*-cells and hence reduce the incidence of diabetes in female NOD mice. The exact mode of action of these effects is presently not known, but the lack of effect on insulitis suggests direct inhibition of the *β*-cell death pathway under the control of IL-1*β*.

## Figures and Tables

**Table 1:** List of antibodies.

Peptide/protein target	Clone	Manufacturer, catalog #	Species	Dilution
CD20		Santa Cruz Biotechnology, sc-7735	Goat polyclonal	1 : 200
CD3	SP7	Abcam, ab16669	Rabbit monoclonal	1 : 100
F4/80	CI:A3-1	Abcam, ab6640	Rat monoclonal	1 : 100
Anti-rat IgG		Vector Laboratories, AI-4001	Rabbit polyclonal	1 : 200
Anti-goat IgG-bio		Jackson ImmunoReserach, 705-065-147	Donkey polyclonal	1 : 200
Glucagon		Phoenix Pharmaceuticals, H-028-02	Rabbit polyclonal	1 : 1000
Somatostatin		Dako, A0566,	Rabbit polyclonal	1 : 1600
Pancreatic polypeptide		EuroProxima, B32-1	Rabbit polyclonal	1 : 1000
Fab2 anti-rabbit-bio		Jackson ImmunoReserach, 711-066-152	Donkey polyclonal	1 : 2000
Insulin		Dako, A0564	Guinea pig polyclonal	1 : 2000
Anti-guinea pig-HRP		Dako, P0141	Rabbit polyclonal	1 : 100

**Table 2:** The effect of diabetic state and compound administration.

	*β*-cell mass		Non-*β*-cell mass		Non-IR endocrine cells	
	*F*-value	*p* value	*F*-value	*p* value	*F*-value	*p* value
Treatment	0.22	0.64	2.25	0.14	1.02	0.32
Diabetic state	20.69	<0.0001	16.74	<0.001	7.52	<0.01
Interaction	0.71	0.41	0.065	0.80	6.26	0.018

	Total endocrine mass		Immune cell mass			
	*F*-value	*p* value	*F*-value	*p* value		

Treatment	0.73	0.40	1.39	0.25		
Diabetic state	17.64	<0.001	5.74	0.02		
Interaction	0.068	0.80	1.34	0.26		

Statistical outcomes of 2 × 2 factorial ANOVA. Results from two-way ANOVA (diabetic state and treatment) are shown with *F*- and *p* values.

## Abbreviations

BG: Blood glucose
NOD: Nonobese diabetic
T1D: Type 1 diabetes
T2D: Type 2 diabetes
Treg: Regulatory T-cell.
